# Heterogeneity in direct oral penicillin challenge protocols in penicillin allergy de-labelling

**DOI:** 10.1016/j.infpip.2021.100185

**Published:** 2021-11-16

**Authors:** Rashmeet Bhogal, Abid Hussain, Louise Savic, Siraj A. Misbah, Ariyur Balaji, Chidanand Hullur, John F. Marriott, Mamidipudi T. Krishna

**Affiliations:** a Department of Pharmacy, University Hospitals Birmingham NHS Foundation Trust, Birmingham UK; b Birmingham Public Health Laboratory, National Infection Service, UK Health Security Agency, Birmingham, UK; c Department of Anaesthesia, Leeds Teaching Hospitals, Leeds UK; d Department of Clinical Immunology, Oxford University Hospitals NHS Trust, Oxford UK; e Department of Acute Medicine, University Hospitals Birmingham NHS Foundation Trust, Birmingham UK; f Department of Anaesthetics, University Hospitals Birmingham NHS Foundation Trust, Birmingham UK; g The School of Pharmacy and Institute of Clinical Sciences, University of Birmingham, Birmingham UK; h Department of Allergy and Immunology, University Hospital Birmingham NHS Foundation Trust, Institute of Immunology & Immunotherapy and Institute of Clinical Sciences, University of Birmingham, Birmingham UK

**Keywords:** Penicillin allergy de-labelling, Antimicrobial stewardship

The rise in antimicrobial resistance (AMR) is a major public health concern [1]. A behavioural shift in antimicrobial stewardship (AMS) is urgently needed to ensure that antibiotics are only prescribed when there is clear evidence of a bacterial infection.

Inaccurate penicillin allergy labels (PALs) are a huge burden on public health and have an adverse impact on AMS [1,2]. Approximately 10–15% of patients present with a PAL, however 90–99% of these labels are deemed inaccurate after a formal assessment by an allergy specialist [[1], [2], [3]].

The aim of a direct oral penicillin challenge (DPC) is to determine if the patient is truly allergic to penicillin. Recent studies have highlighted the safety and feasibility of this intervention in a controlled secondary care environment following a relatively simple process of risk stratification involving a standardised drug allergy history [1,[3], [4], [5], [6], [7], [8], [9], [10]]. Penicillins commonly employed in DPC include amoxicillin, flucloxacillin or phenoxymenthylpenicillin [2,3]. Of these antibiotics, the choice of amoxicillin as a representative member of the penicillin family has particular advantages on account of its widespread use and ability to identify most patients with genuine allergy owing to its core beta-lactam ring and allergenic amino side chain. Other penicillin/beta-lactam antibiotics have also been used. There is considerable heterogeneity in the DPC protocols available [1,[3], [4], [5], [6], [7], [8], [9], [10]]. Patients are usually given a single oral dose either as a bolus or as a graded incremental challenge and monitored for both immediate and delayed onset of symptoms. Some protocols have employed a 3–5 day course of the antibiotic, usually at a sub-therapeutic dose, in an attempt to capture delayed hypersensitivity reactions (HSRs). This is especially important in patients who describe delayed symptoms during the index episode or present with an indeterminate history [3]. Table I summarises some DPC protocols employed for therapeutic and opportunistic de-labelling. Typically, these are observational studies with a relatively small sample size. In most studies, the negative predictive value (NPV) of a DPC was high, ranging between 87%-100%. The majority of positive DPC reactions are delayed, relatively mild and probably represent cutaneous type IV HSRs, which are in favour of employing a prolonged DPC protocol.

**Table I:** Recent studies highlighting heterogeneity in DPC protocols for opportunistic de-labelling

Author	Study type	Adult or Paediatric	Skin testing prior to DPC	Drug	Dose	Type of challenge: STAT dose or graded	Monitoring and follow-up	NPV	Classification of reactions after DPC
Labrosse *et al.* [4] Canada	Prospective study N = 130	Paediatric	No	Amoxicillin	Dose: 45 mg/kg/dose 1/100 Then 1/10 Then full dose Discharged with 4 day course of 45 mg/kg/dose	3 step graded with follow up course of amoxicillin for 4 days	Every 30 minutes Follow- up on day 5 Further follow-up after 2 years of the challenge.	93%	Type I HSR 2.3%: mild urticarial, pruritus and mild localised ear angioedema Type IV HSR 2.3% Mild maculoapular rash 2.3% equivocal
Du Plessis T [5] New Zealand	Prospective interventional study N = 250 DPC N = 34	Adults	No	Amoxicillin	Doses administered every 30 mins Placebo, placebo, 5 mg, 50 mg, 500 mg up to adult dose (1000 mg TDS). If the patient did not need continued treatment, maximum 24 hours was given as part of the challenge.	Graded. If opportunistic de-labelling, challenge continued for 24 hours.	Monitoring not mentioned Follow up at 1 month and after 1 year	91%	Type I HSR – none Type IV HSR (N = 3) mild: ‘itchiness, rash and redness on trunk’.
Savic L *et al.* [6] UK	Feasibility study N = 74 DPC N = 56	Adults	No	Amoxicillin	10%, 50% and 100% of the full dose: 500mg Discharged with 3 days course of antibiotics.	Graded with follow-up course for 3 days	Baseline blood pressure, heart rate and oxygen saturations. Measured again during challenge only if there was a clinical need The patient was observed for 1 hour after the final dose Followed up at day 5–7 after DPC After 3 months, follow up with the GP to check the allergy label	98%	Type I HSR: Urticaria after the second dose Type IV HSR: None 4 patients reported mild non allergic reactions: sore throat and cough, worsening of previously diagnosed arthralgia and mild nausea
Fransson S *et al.* [7] Denmark	Prospective observational study N = 1590 DPC's	Adults	No	Penicillin that caused the index reaction	3 steps: 1:100 dilution, 1:10 dilution, full dose 30–45 minutes apart Or Full therapeutic dose Discharged with 3–10 day course of penicillin as part of the DPC.	Full dose or graded with follow up course for 3–10 days	Patients were monitored for 2 hours after the DPC Follow up after DPC is not mentioned	89%	Penicillin V: Type I HSR: 14 Type IV HSR: 54 Aminopenicillins: Type I HSR: 13 Type IV HSR 66 Other penicillins: Type I HSR: 2 Type IV HSR: 18 Skin and airway reactions were the most common where DPC was positive.
Chambel M *et al.* [8] Portugal	Prospective observational study N = 82 for DPC with the ‘culprit drug’ (beta-lactam)	Paediatrics	For children who refused skin testing, DPC was offered	Penicillin that caused the index reaction	Start at a low^17^ dose and increase to the maximum single dose for the culprit drug every 30 minutes 5 day course prescribed to take home	Graded with follow up course for 5 days	monitoring not mentioned Follow up 48 hours post DPC	87%	Type I HSR: 4 patients Type IV HSR: 1 Unknown: 6 All reactions were mild skin reactions, which occurred 1 hour after the DPC.
Vezir E *et al.* [9] Turkey	Prospective observational study N = 119	Paediatrics	No	Penicillin that caused the index reaction	Amoxicillin–clavulanic acid 40 mg/kg/day divided 2 doses Amoxicillin 40 mg/kg/day divided 2 doses Ampicillin–sulbactam 50 mg/kg/day divided 2 doses Penicillin V 25 mg/kg/day divided 3 doses Discharged with a 5 day course of the penicillin that caused the index reaction	Graded with follow up course for 5 days	Monitored for 2 hours for immediate reactions Follow up after DPC is not mentioned	97%	All reactions were seen with co-amoxiclav All reactions were mild Type I HSR 3 patients: urticaria Type IV HSR 1 patient: urticaria
Trubiano JA *et al.* [10] Australia	Multicentre prospective pilot study N = 46	Adults	No	Penicillin VK Or Amoxicillin	250 mg Choice of penicillin depended on the index reaction ∗For patients with ‘delayed penicillin or amoxicillin hypersensitivity’ 250mg BD for 5 days with the same antibiotic was given.	Full dose with follow up course for 5 days∗	Observed for 2 hours Follow up for 5 days after the DPC	100%	None

From an allergists' perspective, de-labelling with a prolonged course of an antibiotic is likely to enhance the sensitivity and the NPV of a DPC. The optimum duration of the antibiotic course for a DPC protocol, however, has not been established. Fransson *et al.* [7] highlighted that a prolonged DPC is important to exclude delayed HSRs. In their study, 45% of the reactions were seen more than 3 days into the DPC and thereby would not have been identified if a relatively prolonged course had not been prescribed [7].

In patients with a history of a delayed onset reaction occurring on days 1–3 of a treatment course of penicillin, a 3 day DPC course may be sufficient, but in those reporting symptoms on day 5 or after, a 3 day course may be considered inadequate. Furthermore, some patients may not be fully reassured with a prolonged ‘sub-therapeutic dose’ owing to the fear about a reaction occurring at a higher dose.

The majority of studies involving DPCs have used a ‘one size fits all’ approach with respect to employing the same dosing regimen regardless of the time of onset of symptoms of the index episode, making interpretation of published data somewhat challenging [[3], [4], [5], [6], [7], [8], [9], [10]]. A relatively prolonged course of a DPC at a sub-therapeutic dose for opportunistic de-labelling contradicts the basic principles of good AMS: start antibiotics where there is evidence of a bacterial infection, at the ‘right’ dose for the ‘right’ duration. However, it may be argued that the benefits of a DPC in terms of the future use of penicillin [[1], [2], [3], [4], [5], [6], [7], [8], [9], [10]] are likely to outweigh the risks of a DPC for opportunistic de-labelling. In these patients, future adverse effects of second-line inappropriate antimicrobial use on the gut microbiome are potentially prevented. Given the drive towards delivering DPCs by non-specialist clinicians and clinical pharmacists, there is a need for optimisation and standardisation of this intervention [5].

A further gap in the literature is the lack of evidence regarding the potential adverse impact on AMR in the context of a relatively prolonged DPC during opportunistic de-labelling. The risk of AMR is theoretically greater in patients undergoing opportunistic de-labelling; this may be further exaggerated by the duration of the DPC protocol e.g. 5 days *versus* 3 days. In the same context, whilst single dose DPC is expected to have a lower risk of AMR, this needs to be balanced with potentially lower confidence in the NPV of the test. Patients with a PAL usually have a history of receiving alternative antibiotics [[1], [2], [3]] and consequently may have an altered gut microbiome (gut dysbiosis) with a greater risk of AMR. Cumulative doses of multiple antibiotics over prolonged periods may also increase the risk of multi-drug resistant organisms that are difficult to treat with routine antibiotics.

The World Health Organisation (WHO) AWaRe classification categorises antibiotics as ‘access’, ‘watch’ and ‘reserve’. Amoxicillin, flucloxacillin or phenoxymenthylpenicillin are listed under the ‘access’ category of the WHO AWaRe classification. Antibiotics in the ‘reserve’ and ‘watch’ category are more likely to develop AMR (see https://www.who.int/news/item/01-10-2019-who-releases-the-2019-aware-classification-antibiotics). On balance, this may justify the acceptability of employing a prolonged DPC protocol with the ‘access’ group of antibiotics for de-labelling as opposed to a prescription of a ‘watch’ category antibiotic such as a carbapenem.

Further research is needed to optimise and standardise DPC protocols. This should include qualitative studies involving healthcare professionals and patients to gain insight into their perspectives and behaviours. An ideal protocol will involve a dosing regimen that maintains high sensitivity and NPV for DPC in excluding an inaccurate PAL, whilst minimising the risk of AMR. In terms of opportunistic de-labelling, low-risk patients with a suspected history of an immediate reaction should be offered a single dose DPC [2,3]. However, a relatively prolonged DPC might confer advantages in patients with an ‘indeterminate history’ or those reporting delayed onset symptoms [2,3]. Optimisation of prolonged DPC protocols will benefit from prospective studies with respect to establishment of clinical tolerance from re-exposure to a full therapeutic course of penicillin antibiotics post-DPC and investigation of the impact of DPC protocols on the gut microbiome and the risk of AMR.

## Data availability statement (if applicable to the manuscript type)

N/A.
